# Multi3Generation: Multitask, Multilingual, and Multimodal Language Generation

**DOI:** 10.12688/openreseurope.16307.2

**Published:** 2024-05-21

**Authors:** Elena Lloret, Anabela Barreiro, Mehul Bhatt, Alberto Bugarín-Diz, Gianfranco E. Modoni, Max Silberztein, Iacer Calixto, Grazina Korvel, Konstantinos Diamantaras, Alkiviadis Katsalis, Oleksii Turuta, Irene Russo, Aykut Erdem

**Affiliations:** 1 University of Alicante, Alicante,, San Vicente del Raspeig, 03690, Spain; 2 Instituto de Engenharia de Sistemas e Computadores: Investigac¸ao e Desenvolvimento (INESC-ID), Lisboa, Lisboa, Rua Alves Redol, 9, 1000-029, Portugal; 3 CoDesign Lab EU, Örebro University, Örebro, Örebro County, S-701 82, Sweden; 4 Centro Singular de Investigacion en Tecnolox´ıas Intelixentes (CiTIUS), Universidade de Santiago de Compostela, Santiago de Compostela, Galicia, 15782, Spain; 5 Institute of Intelligent Industrial Systems and Technologies for Advanced Manufacturing, National Research Council, STIIMA CNR, Bari, via Lembo 38F, 70124, Italy; 6 Universite de Franche-Comte, Besançon, Bourgogne-Franche-Comté, 30-32, rue Megevand, 25030, France; 7 Amsterdam UMC, Department of Medical Informatics, Amsterdam Public RHealth Research Institute, Amsterdam, Meibergdreef 9, 1105 AZ, The Netherlands; 8 Amsterdam Public Health, Methodology and Mental Health, Amsterdam, North Holland, The Netherlands; 9 Vilnius University, Vilnius, Akademijos st. 4, 08412, Lithuania; 10 Department of Information and Electronic Engineering, International Hellenic University, Thesaloniki, 57400, Greece; 11 Kharkiv National University of Radio Electronics, Ukraine, Nauky Ave, 14, 61166, Ukraine; 12 ILC CNR “A. Zampolli”, Pisa, Via G. Moruzzi, 1, 56124, Italy; 13 Koç University, Istanbul, Sarıyer, 34450 ˙, Turkey

**Keywords:** Natural Language Generation, Language Technologies, Multi3Generation, Multilinguality, Multimodality, Multi-task

## Abstract

The article emphasizes the critical importance of language generation today, particularly focusing on three key aspects: Multitasking, Multilinguality, and Multimodality, which are pivotal for the Natural Language Generation community. It delves into the activities conducted within the Multi3Generation COST Action (CA18231) and discusses current trends and future perspectives in language generation.

## Plain language summary

The Multi3Generation COST Action is a collaborative project that brings together researchers from various fields, all centered around Natural Language Generation. Natural Language Generation involves using computers to generate human-like language for tasks such as translation, summarization, question-answering, and dialogue interaction, among others. The Action addresses common challenges including efficient information representation, advanced machine learning techniques, managing uncertainty in human-Natural Language Generation interactions, and using structured knowledge from diverse sources like databases, images, and videos. Its overarching goal is to make NLG beneficial to society and widely accessible by fostering collaboration between industry and academic experts.

Structured into five working groups, the Action focuses on specific aspects of Natural Language Generation, such as understanding and generating different types of information, developing efficient machine learning algorithms, enhancing dialogue and conversational language generation using knowledge bases, and fostering industry collaboration and end-user engagement. With over 133 scientists from 34 countries involved, spanning disciplines from computer science to linguistics, the project promotes diversity and inclusivity, with 60% male and 40% female participants. Relevant businesses like Unbabel and JabberBrain and other AI stakeholders like the Center for Responsible AI contribute to the Action, aiming to have a broader European impact.

The Multi3Generation Action prioritizes three main areas: Multitasking, Multilinguality, and Multimodality, aiming to enhance language generation in these domains to support underrepresented languages and meet diverse user needs. The article provides insights into the initiatives and planned activities of Multi3Generation, offering valuable information for those interested in NLG and shedding light on future perspectives in this field.

## Disclaimer

The views expressed in this article are those of the author(s). Publication in Open Research Europe does not imply endorsement of the European Commission.

## 1 Introduction

Today, stochastic-, probabilistic-, statistical- or neural-network-based methods are trendy, thanks partly to OpenAI’s ChatGPT ^1^, Google’s Bard ^2^, and Microsoft’s Sydney ^3^ chatbots which have been garnering a lot of attention for their ability to generate detailed answers across many knowledge domains. Consequently, most researchers in Natural Language Processing (NLP) develop systems that use stochastic methods to extract solutions from massive databases used as “cheat sheets” instead of trying to formalize what meaning needs to be produced and in what style. Moreover, NLP software that uses training corpora associated with stochastic methods is being used daily: people regularly talk to their smartphone thanks to OK Google or Apple Siri, to their home thanks to Amazon’s Alexa, and use Machine Translation applications such as Google Translate, Deepl or ChatGPT Translator Plus routinely for their personal and business needs. Natural Language Processing (NLP) addresses all computational and linguistic aspects necessary to address both the comprehension (also known as Natural Language Understanding – NLU) and generation of language ( *i.e.*, Natural Language Generation – NLG) ^1^. In fact, the emergence of deep learning based approaches has significantly revolutionized the traditional conception of NLG ^2^, thus making it more popular not only for the scientific community but also attracting the attention of industry and society.

The COST Action Multi3Generation (CA18231), coordinated by INESC-ID ( *Instituto de Engenharia de Sistemas e Computadores: Investigação e Desenvolvimento em Lisboa*), started in September 2019, aims at fostering an interdisciplinary network of research groups working on different aspects of NLG. The Action frames NLG broadly as the set of tasks where the ultimate goal involves generating language, such as machine translation, question answering, summarization, simplification, or dialogue generation, to name just a few. In fact, the emergence of Generative Artificial Intelligence (AI) ( *e.g.* ChatGPT) has significantly revolutionised the traditional conception of NLG ^2^, thus making it more popular not only for the scientific community but also attracting the attention of industry and society. Regardless of the NLG application to address, there are a set of common aspects that are critical for the successful development of the different applications, which constitute the core challenges Multi3Generation focuses on. These are:

- *Data and information representations.* More and more, inputs to NLG applications are heterogeneous and varied, coming from different sources ( *i.e*., tabular data, text, images, videos, Knowledge Bases (KB) or graphs).
- *Machine Learning (ML).* How can modern ML methods such as multitask learning (MTL), representation learning and structured prediction be leveraged for NLG?
- *Interaction.* Applications of NLG, *e.g.*, Dialogue Systems, Conversational Search Interfaces and Human-Robot Interaction, pose additional challenges to NLG due to uncertainty derived from the changing environment and the non-deterministic fashion of interaction.
- *KB exploitation.* Structured knowledge is key to NLG, supporting ML methods that require expansion, filtering, disambiguation, or user adaptation of generated content. In this respect, how can we efficiently exploit commonsense, world knowledge and multimodal information from various inputs such as knowledge bases, images, and videos to address NLG tasks?

In addition, and due to the numerous applications that can be derived from NLG, it is of great importance that they are beneficial to society. For this, the industry must play an important role together with academia, which results in making NLG available to the general public. In this respect, language generation applications, such as machine translators, summarizers, paraphrasers, conversational agents, *etc.*, may become essential tools on a daily basis.

In order to face the aforementioned challenges, Multi3Generation is organized into five Working Groups (WG), where each focuses on, and tries to advance research in a specific aspect of NLG. In particular, these are:

- WG1 – Grounded Multimodal Reasoning and Generation
- WG2 – Efficient Machine Learning Algorithms, Methods, and Applications to Language Generation
- WG3 – Dialogue, Interaction and Conversational Language Generation Applications
- WG4 – Exploiting Large Knowledge Bases and Graphs
- WG5 – Industry and End-User Liaison

The more than 133 participants in the Action, belonging to 34 different countries, include experts in computer science, data science, linguistics, computational linguistics, and digital humanities. This multidisciplinary and interdisciplinary expertise has greatly benefited Multi3Generation, as challenges have been addressed and solved from different angles. With respect to gender dimension, there are more men than women participating in Multi3Generation. The percentages are 60% men *versus.* 40% women. To ensure a more significant impact on a European scale, several relevant companies related to NLG technologies are also involved, such as the machine translation company, Unbabel ^4^ and the startup JabberBrain ^5^.

In order to achieve the objectives of Multi3Generation, different types of activities and dissemination events are developed:

- *Meetings.* The aim of this type of activity is to support Multi3Generation scientific and networking activities in line with the objectives specified in the project.
- *Short-term scientific missions (STMS).* These are institutional visits aiming to support individual mobility, fostering collaboration between individuals. They aim to create synergies between researchers to encourage joint research and maximize the results of the project.
- *Training schools.* The purpose of training schools is to facilitate capacity building on NLG, specifically targeted to early careers investigators ( *i.e.*, those individuals who obtained their PhD/doctorate up to eight years ago) and PhD students. Both the Training Schools that took place in 2022 on “Representation Mediated Multimodality” ^6^ and on “Creative Natural Language Generation” ^7^ are examples of initiatives conducted in this category.
- *Workshops.* Workshops aim at attracting the research community’s attention in the specific topics related to Multi3Generation. The first Workshop on Multilingual, Multimodal and Multitask Language Generation ^8^ that will be co-located with ‘The 24th Annual Conference of The European Association for Machine Translation” (EAMT 2023), the first Symposium on Challenges for Natural Language Processing ^9^ (CNLPS’23), co-located at the 18th Conference on Computer Science and Intelligence Systems (FedCSIS 2023), or the Workshop on Multimodal, Multilingual Natural Language Generation and Multilingual WebNLG Challenge ^10^, co-located at the 16th International Natural Language Generation Conference (INLG 2023), are some examples of conducted activities under this category.
- *Participation in broader events.* This type of activity is focused on disseminating the Multi3Generation Action within the research community. Examples of initiatives conducted within Multi3Generation can be found on the website ^11^.

This article is an opportunity for anyone interested in NLG to discover the initiatives carried out within Multi3Generation COST Action and benefit from the ongoing and coming planned activities.

## 2 Natural Language Generation in the context of Multi3Generation

In contrast to the more classical definition of NLG, where four main stages are involved in the process, the ones of content determination, macroplanning, microplanning, and surface realization and each one can be addressed using different approaches ^3^, the emergence of deep learning architectures, such as Transformers ^4^, have brought in integrated architectures, where the whole NLG is tackled at a once, without any distinction within the aforementioned stages. These new approaches require including issues not concerned with language generation in an immediate sense, but that could obviously inform or improve language generation models, as for instance, pre-trained models, transfer learning or parameter estimation ^5– 7^.

Multi3Generation compiles expertise from different fields, such as computer science, in particular, Natural Language Processing and Artificial Intelligence, humanities, including digital humanities, or mathematics, so the joint research efforts and synergies contribute to putting Europe’s research at the forefront of the NLG field.

### 2.1 Why these three dimensions: multitasking, multilinguality and multimodality?

Multi3Generation focuses on three dimensions: (i) multitask, (ii) multilinguality and (iii) multimodality. The importance of these dimensions for language generation is in line with four of the most well-known V’s of Big Data: volume, velocity, veracity, and variety ^8^, and more specifically to variety dimension. There is no doubt that digital information is increasing exponentially as far as volume and velocity are concerned. Variety is important for language generation, since in the current context, most of the data generated today (around 80%–90%) is in an unstructured manner ^12^, thus leading to heterogeneous data in different formats and modalities such as audio, video, text, concepts, Resource Description Framework (RDF) triples, numeric data without forgetting that in the case of audio and text, information in different languages can be also found.

Focusing on the specific dimensions addressed in this COST Action, multilinguality is a central goal if machines are to perform seamless language generation. End-users are in diverse communities, including linguistic communities, and not all languages are equally represented in the digital landscape, as studied by the “Endangered Languages Project” ^13^. As proof that multilinguality is important within European policies, there is an ongoing initiative for developing an agenda and a roadmap for achieving full digital language equality in Europe by 2030 ^14^.

Multi3Generation emphasizes multilinguality in generation, including European languages with a small number of speakers ( *e.g.* Welsh, Sami), which are also well-represented in the Consortium, as well as languages with low resources from a languages technologies point of view. Under-resourced languages would benefit from advances in multilingual, multitask and transfer learning (which raise the possibility of exploiting large-scale resources for neighboring languages to the benefit of small languages for which training data is harder to come by). The project “DIALECT – Natural Language Understanding for non-standard languages and dialects”, led by Prof. Barbara Plank ^15^, MC member of Multi3Generation also addresses the topic of processing languages underrepresented in the Internet. We can include here undergoing projects, such as “eSPERTo – System for Paraphrasing in Editing and Revision of Texts ^9, 10 16^, and “Paraphrasary” (Portuguese version “Parafrasário”) ^11^, both led by the Action’s Chair, Dr. Anabela Barreiro, developed for Portuguese as part of a larger multilingual generation project involving paraphrasing and translation, for which several experimental results have been already published ^12– 15^.

Another important dimension to take into consideration is multimodality. As stated in 16, 17, multimodality refers to “Our experience of the world is multimodal - we see objects, hear sounds, feel the texture, smell odors, and taste flavors. Modality refers to the way in which something happens or is experienced”. In the context of Multi3Generation we address text, vision, and speech. Finally, multitask refers to the multiple application tasks that can be directly derived from NLG, including, among others, translating from one language to another, describing, asking or answering questions from visual content, such as images or videos, summarizing, or simplifying text.

## 3 Key aspects for Multimodal Natural Language Generation

This section explains the different working groups within Multi3Generation, also discussing some key facts/activities developed in them. WG5, which is related to industry and society will be later discussed in Section 6, due to the importance and popularity that NLG is recently having.

### 3.1 Grounded Multimodal Reasoning and Generation – WG1

Linguistic expressions and/or relational categorisations are called grounded when they are linked to non-linguistic, especially quantitative perceptual data, such as information coming from modalities such as vision and audition in spacetime. Such perceptual data could pertain to, for instance, dynamic spatio-temporal phenomena both in an embodied as well as disembodied interaction context. Grounding is, in essence, a key aspect of semiotic construction, *e.g.*, enabling high-level meaning acquisition, analogy, and has been a long-standing challenge in Artificial Intelligence and related disciplines. WG1 particularly focuses on grounded knowledge representation, reasoning, and learning (for linguistic purposes) through the following emerging research areas ^18, 19^:

- Space and Language
- Computational Linguistics
- Cognitive Linguistics
- Cognitive Vision
- Vision and Language
- Neurosymbolism
- Narrative - Discourse
- Computational Models of Narrative
- Speech and Language Technologies
- Human-Centred AI

With a categorical focus on the use of multimodal information to reason, learn, and generate natural language, central themes addressed in WG1 include:

- Explainability and transparency in multimodal models
- Neurosymbolism / Interaction between symbolic & sub-symbolic representations in models
- Commonsense knowledge representation and reasoning, and its role in multimodal models
- Complementarity / redundancy among data sources or modalities
- Situated reasoning, e.g., for language generation, decision-making

The primary focus on WG1 has been on investigating the significance of *grounded* knowledge representation and reasoning as a foundational basis to mediate multimodal perception and interpretable sensemaking (for language generation, amongst other things) through deeply semantic (neurosymbolic) characterizations supporting explainable commonsense reasoning and learning ^18– 21^. WG1 has also been involved in drawing up standards for multimodal data sources, and defining a research roadmap through an appraisal of existing work and identifying gaps to be addressed in future work. Some representative research outputs from collaborations within WG1 include Vision And Language Structured Evaluation (VALSE) , where researchers propose a shift in how to evaluate vision-and-language models. Instead of focusing on standard task-based evaluations ( *e.g.*, assessing the accuracy of a model on visual question answering), they propose to systematically assess model behavior by measuring how well models encode specific targeted linguistic phenomena. For that purpose, authors compare model behavior in the presence of correct *versus*. counterfactual examples. The phenomena studied include whether models understand the presence/absence of a certain object type in an image ( *i.e*., existential statements), whether models distinguish the presence of one vs. many instances of a specific object type in an image ( *i.e.*, plurality), whether models can count the number of instances of a particular object type in an image ( *i.e*., counting), among other *visuo-linguistic capabilities*. Work in WG1 has resulted in the Multi30K dataset, a parallel corpus of the multimodal dataset for the Ukrainian language ^22^. Currently, image descriptions are available in English, German, French, Czech, Turkish, and Ukrainian.

WG1 has also developed, in close collaboration with WG2 and WG3, a flagship Training School (TS), first organized in Germany in 2022. The Training School 2022 on *Representation Mediated Multimodality* ^17^ provides a consolidated perspective on the theoretical, methodological, and applied understanding of representation mediated multimodal sensemaking at the interface of language, knowledge representation and reasoning, and visuo-auditory computing. Intended purposes -addressed in the school- encompass diverse operative needs such as explainable multimodal commonsense understanding, multimodal generation/synthesis for communication, multimodal summarization, multimodal interpretation guided decision-support, adaptation & autonomy, and analytical visualization. In addition to hosting an invited faculty delivering lectures, intensive tutorials, and keynotes, the school also provides opportunities for young researchers to position ongoing/early stage research, discuss, and network with school faculty and participants.

### 3.2 Efficient Machine Learning algorithms, methods, and applications to language generation – WG2

The current state-of-the-art NLG systems are all based on deep learning and utilize different neural network architectures, most of which are based on the Transformer architecture. These models also differ from each other based on the learning strategies they employ, such as multitask learning, transfer learning or pre-training, structured prediction, and different types of generative models. WG2 specifically deals with the efficient machine learning techniques behind these recently proposed neural NLG models. Another key focus is the integration strategies for multimodal data, as the input for an NLG system can involve a variety of formats, including not just text but other forms of structured data like databases, images, plots, audio, or video.

The discussions within the workgroup resulted in the survey titled “Neural Natural Language Generation” ^17^, which focuses on the most common NLG applications, namely *machine translation*, *description generation*, *abstractive summarization*, *automatic speech recognition*, *text simplification*, *question generation* and *visual question generation*, and *dialog generation*. This survey analyzes the maturity level of each NLG task with respect to the dimensions of *multilinguality*, *multimodality*, *learning strategies* and *controllability*, and draws some conclusions about the remaining challenges and possible future work, providing a more holistic perspective regarding the aforementioned four dimensions. Derived as a direct outcome of this survey, a curated list of resources on Neural NLG, focusing on multilinguality, multimodality, controllability and learning, is publicly available ^18^.

Furthermore, another research line being investigated within Multi3Generation project is the detection and integration of commonsense knowledge to any downstream NLG task, such as question & answering, abstractive text summarization. COMET ^23^ and KG-BART ^24^ were initially chosen to approaches that could be helpful for this aim.

KG-BART tackles the generative commonsense reasoning task using the CommonGen ^25^ dataset. The task is to generate natural language sentence based on common sense using as input concepts-words. It is based on BART model ^26^ and uses commonsense knowledge from ConceptNet ^27^. Knowledge base as augmented knowledge. In the same level, COMmonsEnse Transformers (COMET) ^28^ can generate new knowledge by adding new commonsense nodes of existing commonsense knowledge graphs like ConceptNet and ATOMIC ^29^ Retrieval Augmented Generation (RAG) ^30^ uses BART transformer-based model for NLG tasks using an extra intermediate step by adding external knowledge with Dense Passage Retrieval (DPR) ^31^. LETO ^32^ is a system of GPLSI lab in Alicante that collects knowledge and creates a Knowledge Graph (KG) with external knowledge from structured and unstructured text where commonsense knowledge could give added value. The outcome of this research line, framed within WG2, but having also tight connections to WG1 and WG4, could be listed as follows:

- Use commonsense knowledge in natural language text (output of KG-BART) in DPR part of RAG model.
- Make use of COMET and KG-BART to generate new knowledge.
- Tweak KG-BART by adding more knowledge (use Comet Transformer) information except the ConceptNet KG.
- Fine-Tune BERT/BART model in relation prediction task using LETO Knowledge Graph and CommonSense KGs and with those updated embeddings use the BERT/BART to NLG tasks.
- After experiments, the aim is to make a multilingual/cross-lingual framework for Natural Language Generation tasks.

Furthermore, incorporating commonsense knowledge into machine learning models improves models’ ability to understand and produce natural language by providing background information and context that is not explicitly mentioned in the text. One of the ways to incorporate knowledge graphs into machine learning models is by using Graph-tosequence (Graph2Seq) models. In this case, the database containing common sense knowledge is a knowledge graph where entities are represented as nodes, and relationships between entities are represented as edges. The issues related to encoding the graph structure into a vector representation and integrating it with existing models are widely discussed within this working group.

### 3.3 Dialogue, interaction and conversational language generation applications – WG3

WG3 focuses on Human-Computer Interaction (HCI) tasks in multilingual and multimodal scenarios applying NLG models to distinct use cases, such as conversational agents, with three main directions:

- New answer generation techniques where the (human) agent will receive suggestions
- New techniques for conversational quality estimation and sentiment analysis
- Creation of multilingual datasets for low-resource languages

The main challenges for WG3 concerns:

- Scarcity of multimodal and multilingual datasets for chat in general.
- Scarcity of multilingual datasets for low resourced languages.
- How to benchmarking the models applied.
- New metrics for multilingual conversational dialogues.

Therefore, this working group works on reviewing the existing literature and resources to conduct: (i) a survey on affective agents and answer generation adaptations to chat data; (ii) a survey on metrics for dialogue systems; (iii) a report on available multilingual datasets for conversational data; and (iv) creating multilingual datasets for low resource languages. Thus, this working group contributes to the cross-task roadmap of the project to responsible AI initiatives since we are working with modalities with severe ethical aspects.

### 3.4 Exploiting large knowledge bases and graphs – WG4

WG4 focuses on using knowledge bases (KBs) and knowledge graphs in NLG, especially for integrating commonsense knowledge and world knowledge into the generated output. An expected result of WG4 is to increase the varieties of knowledge resources and language resources used in NLG. Therefore, WG4 analyzes how to efficiently integrate multimodal KBs, considering theoretical models of semantics and semantic processing that can accommodate linguistic and perceptual information. Among the aims of this working group, increasing existing data-to-text NLG training sets with multilingual and multimodal content and testing neural NLG models performance on psycholinguistic datasets were listed as relevant for its members. However, during the recurrent meetings and a training school about creative natural language generation held in Venice in September 2022, the evaluation of the generated outputs, especially when characterized by multimodality and creativity as in text-to-image neural systems, made the categorizations and the empirical findings of psycholinguistics relevant. In particular, a debate about the features of artificially created outputs such as texts and images made clear that new categorizations and annotation experiments are needed to properly understand how artificial creative artifacts are perceived.

One concrete example of integrating knowledge graphs into NLG was described in a paper presented at the 13th Conference on “Data analysis methods for software systems” ^33^. More specifically, the paper reported how sequence-to-sequence (Seq2Seq) learning, which is a widely used encoder-decoder paradigm in natural language generation, can be improved by incorporating graph neural network (GNN) techniques to address challenging issues such as long-dependency problems. Graph-to-sequence (Graph2Seq) GNNs-based encoder-decoder models are increasingly discussed in the literature for tackling particular tasks. Graph2Seq models have shown superior performance compared to Seq2Seq models in various tasks, including neural machine translation, text summarization, and question generation. Disregarding success, Graph2Seq models also inherit challenges, for example, encoding relations between distant nodes. The outstanding issue is how to incorporate text information and transform it into a graph. The hypothesis put forward by the members of this working group in collaboration with the members of WG2 was the following: enriching the graphs with external knowledge and testing pre-trained language models can benefit text generation tasks; using sentence transformers, the KGs can be enriched with extra relationships between available entities in the dataset; transformation of the title from text to the graph can also be beneficial. These hypotheses were validated using the following experimental set-up: the similarities between entities not present in the existing KG of Abstract GENeration DAtaset (AGENDA) dataset ^19 34^ were found; BART-large pre-trained language model was fine-tuned in the abstract generation problem.

## 4 A novel methodology for text generation

In the framework of the COST’s Multi3Generation Action, we organized an intensive training school ^20^ (class + lab) in which we showed the limitations of the stochastic approaches, and presented an alternative methodology to develop automatic generators, automatic paraphrase generators, and machine translation systems. The basis of this methodology is to formalize every level of the linguistic phenomena involved during the text generation process. For instance, such a system must be able to produce an English sentence that represents the predicate “Joe loves Lea” in the past, aspect +Stop, intensive +High, focus on “Joe”, and “Lea” pronominalized, that is “ *It is Joe who stopped being madly in love with her*”. Such a system must be able to access linguistic databases that know what aspectual verbs are available in English, how to express intensivity for love, how to pronominalize an object, *etc.*

During the training course, we have described each of the linguistic phenomena that must be considered to construct such a system, including:

- inflectional morphology ( *e.g.*, to stop → stopped)
- derivational morphology ( *e.g*., to love → to be in love with)
- local syntax ( *e.g*., Joe loves Lea → it is Joe who loves Lea)
- coreference ( *e.g.*, Joe loves Lea → Joe loves her)
- intensifier ( *e.g*., to be in love → to be madly in love)

One characteristic of the novel methodology presented is that all its linguistic formalization is neutral, *i.e.*, it can be used both to parse an existing text and to generate a new text. That makes these linguistic resources suitable not only to develop automatic text generators, but also to parse a text and re-generate it with some different values, such as tense, aspect, modality, or intensifier, *i.e*., to paraphrase them. Moreover, because there exist linguistic resources in the same format for over 30 languages, it is a matter of parametrizing the application to parse a text in one language and then generate its “paraphrase” in another language, that is, this has allowed us to develop machine translation applications.

The workshop was split into two sessions:

- A theoretical session where the methodology was presented as well as the various types of linguistic resources involved during text generation/paraphrasing/translation.
- A hands-on session that showed the participants how to build the crucial components and linguistic resources for such a system, using the NooJ linguistic development environment ^21^.

## 5 Tools and resources to paraphrase and translation Generation

For NLG, it is crucially important the quality of the data that is used in the generation task, but also the tools that are used, how they were built, their strengths and limitations, the ability for the human to control the process, curate, and improve the quality of a generative system. Most systems are black boxes, built in a way in which humans do not have control over the generative process. Some paraphrases generation or extraction techniques may simply involve semi-automated procedures, while others may consist of supervised alignment trained in manual alignments (used for monolingual or bilingual term extraction). Even when supervised training is used in building a system, at a particular stage, the process may get out of human control. We search for ways of developing glass-box systems having in mind the “human-in-the-loop”, that is, the human in control of the system from the very first stage of its development.

Some research has been done at INESC-ID Lisboa in the development of tools and resources to generate paraphrases and translation. Within the Multi3Generation COST Action further developments to initial fundamental research has been recently done, namely the creation of novel resources named ‘paraphrasaries’, which are complex complementary extensions of dictionaries, designed to be used in monolingual or multilingual applications ^11, 35^. The concept of “paraphrasary”, akin to a dictionary at a multiword level, appeared to fill in a void in the creation of linguistically more sophisticated resources. With regards to MT, it is not possible to achieve quality translation without involving comparable quality paraphrasing knowledge and skills (capabilities), because paraphrases are essential for implanting semantic knowledge edge to ensure high fidelity translation instead of approximate or good enough translation. We trust that it is important to revisit alignment tools and methodologies and to define collaboratively-built guidelines for alignment of paraphrases and translation, defining and measuring different degrees of equivalence, while increasing the volume of paraphrastic units, *i.e.*, pairs of alignments that match semantically identical or similar units of meaning, not only in commonly used corpora, but extend the process to more creative types of text.

## 6 Impact of Multi3Generation and perspectives for the future of LG – WG5

Several European industries and markets can benefit from the outcomes of this Action, including e-commerce (the ability to translate multi-modal content and, as a result, reach wider audiences), and customer services (through automated assistants). In addition, industries can benefit from Multi3Generation through the development of innovative human-machine Interactions. In this regard, it should be noted that the European modern industry is leaning toward Industry 5.0 which foresees the centrality of the worker in the industrial system ^36^. In this new scenario, the interactions between humans and machines (and, in particular, robots) are evolving to bring advantages in terms of efficiency, ergonomics, flexibility, and safety ^37^.

One of the key features for the evolution of human-machine interactions is communication which can be implemented through the use of NLG. In fact, the latter allows generating written or oral content to give the machine the ability to effectively communicate ^38^, thus creating the conditions to realize real applications of human-machine co-working. For instance, it is possible to integrate a human-robot interaction in an assembly process, leveraging NLG and ML to give precise instructions to the operator according to their personal characteristics. This can also be achieved using tailormade chatbots and conversational search Interfaces, which read information about the process and act accordingly.

Along with the opportunities, the evolution of the human-machine interaction towards Industry 5.0 ^22^ brings several challenges, both technical and human-related ^39^. In particular, NLG-related challenges mainly concern the fluidity and flexibility of the communication and the quality of the generated content ^38^. Moreover, among the challenges, it should also be reported the analysis of the ethical repercussions of the adoption of these technologies. In particular, some aspects that should be taken into account are the generation of harmful content or content that can be used to violate the law and the generation and spreading of fake news and misinformation.

Regarding challenges and future perspectives for language generation, we should consider, in the first place, the two main existing approaches for NLG systems, as indicated in Section 2: more traditional rule-based approaches, which mostly follow the stages pipeline proposed by 40 and more recent End-To-End (E2E) approaches based on the attention mechanism and the Transformer-based models, *e.g.* BERT ^41^, GPT-2 ^42^, or GPT-3 ^43^. Both approaches present high scientific and technological challenges and clear needs to develop computational and linguistic resources for building impactful NLG-based systems and applications.

Although E2E approaches have burst recently, rule-based generation is still a valid approach, since it is based in a careful design which, in many cases, is the only option for achieving reliable applications ( *e.g.*, critical applications where reliability is a relevant requirement). The challenge here is that computational resources must be created or improved to automate and facilitate the design process and/or improve the fluidity of the generated texts. In this sense, valuable resources such as the realization libraries SimpleNLG-GL ^44^ or others should be improved in terms of their efficiency, abstraction, generalization of structures, vocabulary, alternate realizations or paraphrases, among others.

On the other hand, E2E models, in general, improve the fluency of the texts when compared to a rule-based model. But, very often, they may suffer from “hallucinations” in which the generated text includes contents which are unrelated to the input data or directly wrong or misleading. The search for an effective method that can foresee, detect, or remedy these situations in NLG E2E models is a major challenge ^45^, as it affects the reliability of these systems, especially in critical or sensitive applications, *e.g*., in which the moral, mental and physical integrity of people are protected.

Furthermore, to train the E2E models, it is necessary to have high-quality corpora which, in some areas are hardly available (this is the usual case in data-to-text systems, for instance). Another scientific challenge of extraordinary interest and relevance is the validation and testing of E2E models. There is currently an intense debate in the scientific community about the generalized use of inadequate automatic evaluation metrics in E2E training, and their lack of sensitivity to the problem of hallucination in texts, and their lack of correlation to human (expert) validations. Manual validations by humans may be a solution for small-scale problems, but not for problems where the variety of texts generated is very high, and may also subject to reproducibility issues ^46^. In this sense, some of the open challenges in this area are:

- New automatic metrics that consider the problems currently existing in E2E generation and methodological strategies in validation that ensure the representativeness of the test sets or samples evaluated by human experts.
- Automated fact-checking mechanisms to prevent or assess the quality and appropriateness of the generated texts against the input data.

Another challenge in the field of rule-based NLG is the fluidity of the generated texts. The definition of rigid structures causes the generated texts to be very similar, becoming unnatural when reading several texts in succession. The inclusion of rules, synonyms, and paraphrases is still insufficient, and therefore work remains to be done in this area for improving the fluidity and readability of generated texts. In this sense, since one of the main objectives of the NLG is to produce texts that are as similar as possible to human texts, it is essential to create automated resources that provide the generated texts with naturalness and fluency, while ensuring that they correctly describe the data requested from the model. For this, the Nooj framework has been explored within Multi3Generation ^47^. Striking a balance between naturalness, linguistic variety, readability and, at the same time, consistency with respect to the data is a difficult task, but also essential for a complete NLG system. Dealing effectively with the problems of scalability and maintenance of this approach is also a major technological challenge.

The hybridization of the two approaches, orienting the E2E systems not to implement the full pipeline, but to the learning of models ( *e.g.*, instances of a grammar), opens a very promising way to improve the quality of texts, the scalability of the approaches and the problem of hallucinations, among others. In Data-To-Text systems, in critical applications, for instance, E2E can be used for simplifying and improving significantly the final linguistic realization stages (merging, for instance, planning and surface realization), whilst content determination is tackled with more reliable classical approaches.

Likewise, taking inspiration from the field of machine translation, a major challenge is to consider the “human in the loop” perspective to orientate the creation of texts not towards a fully automated successful final realization, but to the creation of texts with an acceptable level of quality and which facilitate the final post-editing by a human operator who provides the result with the expected quality and precision.

In order to investigate the practical exploitation of the main outcomes of Multi3Generation COST Action, also involving industrial stakeholders, it is worth introducing WG5, whose activities are described in the following subsection.

Working Group 5 “Industry and End-User Liaison” aims to develop links with industry and end-users. As such, its activities embrace collaboration between academic and industrial partners, both on academic projects and real-world product development, seeking to stimulate ideas for novel cross-modal applications.

In addition to having participants from the industry, company stakeholders are welcome to the Industrial Advisory Board, which has the functions of advising and informing the Management Committee’s activities, and fostering collaboration between industrial and non-industrial participants, including placements for Early Career Investigators and co-organization of shared tasks including the construction of benchmark datasets. Furthermore, WG5 aims to coordinate user requirement surveys and other methods for obtaining end-user input. In this regard, the potential of the technologies dealing with the action is investigated according to two different activities:

1. Conducting a survey among different industrial and academic stakeholders (involving at the beginning the MC members). The survey will have two different perspectives:
  (a) Eliciting requirements from end-users;
  (b) Reporting the available industrial data sources ( *e.g.* ontologies) which can be used to feed NLG tools;
2. analyzing the state of the art with the aim to identify and classify NLG-based applications already available in the industry ( *e.g.*, such as conversational search interfaces; grounded dialogue models; real-time dialogue models; and conversational robots).

The two above activities are currently ongoing. At the end of these activities, the main achieved outcomes will be reported within specific reports.

## Conclusions

There is no doubt that NLG technology has increased in popularity in the last few years. Although its successes, we can also find some limitations and risks associated to the misuse of such potential technologies. Multi3Generation COST Actions brings together researchers whose expertise is related to different angles of NLG. With the joint efforts of the whole Multi3Generation community, relevant progress has been made while the ongoing of the Action, contributing to putting Europe at the forefront of (human-centered) NLG research. In this paper, an overview of Multi3Generation COST Action is provided, discussing also its WGs, activities, and outcomes obtained. Despite the fact that Action will finish in March, 8th 2024, we expect sustained collaborations between Multi3Generation members either in future COST Actions or in other research initiatives. We also plan to continue jointly developed dissemination actions such as training schools.

## Ethics and consent

Ethical approval and consent were not required

## Data Availability

No data are associated with this article.
