# Supplementary material for: Multi3Generation: Multitask, Multilingual, and Multimodal Language Generation
Source: Open Res Eur. 2024 May 21;3:176. Originally published 2023 Oct 12. [Version 2] doi: 10.12688/openreseurope.16307.2 (PMC10733662; doi:10.12688/openreseurope.16307.2)
Supplement: Peer review history

# Reviewer report: Reviewer response for version 1

**DOI:** 10.21956/openreseurope.17604.r36459

**Published:** 2023-12-20

**Authors:** Marco Polignano

**Affiliations:** 1 Department of Computer Science, University of Bari 'Aldo Moro', Bari, Italy

The authors present a scientific paper that provides a comprehensive overview of the activities conducted within the framework of COST Action Multi3Generation (CA18231). The contribution is easy to read and understand and well supports the motivation behind the idea of the action. The contributions made throughout the project are both numerous and noteworthy, frequently finding dissemination through prestigious conferences and esteemed scientific journals within the relevant field.

 To enhance the efficacy of communicating the results obtained from the action, I recommend offering a more detailed exposition of the created artifacts and their corresponding identification procedures within the publicly accessible GitHub repository. This additional information would facilitate a clearer understanding of the artifacts and enable researchers to effectively utilize and build upon the outcomes of the project. Furthermore, it would be advantageous to provide a concise summary of select scientific achievements, supplemented by accompanying experimental results.

 This would allow readers to grasp the significance of the research conducted and facilitate future exploration and replication of the findings.

 In conclusion, the contribution presented in this work is meticulously crafted, exhibiting a high level of proficiency in both content and presentation. The thoroughness of the research, the clarity of the writing, and the potential impact of the findings within the scientific community make it a valuable contribution.

Where applicable, are recommendations and next steps explained clearly for others to follow? (Please consider whether others in the research community would be able to implement guidelines or recommendations and/or constructively engage in the debate)

Yes

Does the article adequately reference differing views and opinions?

Yes

Are all factual statements correct, and are statements and arguments made adequately supported by citations?

Yes

Is the rationale for the Open Letter provided in sufficient detail? (Please consider whether existing challenges in the field are outlined clearly and whether the purpose of the letter is explained)

Yes

Is the Open Letter written in accessible language? (Please consider whether all subject-specific terms, concepts and abbreviations are explained)

Yes

Reviewer Expertise:

Natureal Language Processing, Artificial Intelligence, Text Generation, Recommender Systems

I confirm that I have read this submission and believe that I have an appropriate level of expertise to confirm that it is of an acceptable scientific standard.

# Reviewer report: Reviewer response for version 1

**DOI:** 10.21956/openreseurope.17604.r35589

**Published:** 2023-11-02

**Authors:** Eugenio Martínez-Cámara

**Affiliations:** 1 Computer Science Department, Universidad de Jaen, Jaén, Andalusia, Spain

The paper presents the relevance of natural language generation and the development of its challenges in the COST Action Multi3Generation.

 From my point of view, the paper presents the progress of the project, and how the consortium is facing the challenges of natural language generation. Likewise, the project also is developing teaching and dissemination activities through the organization of workshops, which is very positive.

 To sum up, I consider that the paper should be accepted for indexing in its current form.

Where applicable, are recommendations and next steps explained clearly for others to follow? (Please consider whether others in the research community would be able to implement guidelines or recommendations and/or constructively engage in the debate)

Yes

Does the article adequately reference differing views and opinions?

Yes

Are all factual statements correct, and are statements and arguments made adequately supported by citations?

Yes

Is the rationale for the Open Letter provided in sufficient detail? (Please consider whether existing challenges in the field are outlined clearly and whether the purpose of the letter is explained)

Yes

Is the Open Letter written in accessible language? (Please consider whether all subject-specific terms, concepts and abbreviations are explained)

Yes

Reviewer Expertise:

Natural Language Processing

I confirm that I have read this submission and believe that I have an appropriate level of expertise to confirm that it is of an acceptable scientific standard.

# Response

**Published:** 2023-11-06

**Authors:** Elena Lloret

**Affiliations:** Software and Computing Systems, Universidad de Alicante, San Vicente del Raspeig, Alicante, Spain

Dear Eugenio, thank you very much for reviewing our manuscript and for finding it interesting to the scope of ORE. Natural Language Generation (NLG) has gained popularity in recent years, partly due to the advancements in Generative AI, so from the Multi3generation COST Action, we believe that is important to provide visibility to the activities conducted within the action,  as well as to provide insights, prospects and challenges related to NLG.
